# *Helicobacter pylori* and Immunocompromised Children

**DOI:** 10.3201/eid1201.050500

**Published:** 2006-01

**Authors:** Prakaimuk Nutpho, Nuthapong Ukarapol

**Affiliations:** * Chiang Mai University, Chiang Mai, Thailand

**Keywords:** Helicobacter pylori, immunocompromised children, prevalence, letter

**To the Editor:** *Helicobacter pylori* has been classified as a carcinogenic pathogen. Its prevalence is high in developing countries. Apart from the known gastrointestinal pathologic changes caused by this organism, reports on the association between *H. pylori* infection and extragastrointestinal diseases have been increasing. Although impaired host immunity should be associated with a high prevalence of this infection, a definitive relationship has not been established. We conducted a cross-sectional study to determine the prevalence of *H. pylori* infection in immunocompromised Thai children.

The study was reviewed and approved by the research ethic committee of Chiang Mai University. From 2003 to 2004, a total of 60 children <18 years of age, who received corticosteroids, immunosuppressive drugs, or both, were enrolled consecutively into this study. Patients who had taken proton pump inhibitors and antimicrobial drugs 2 weeks before the study began were excluded. Stool specimens were collected and immediately stored at –20°C before analysis with the *H. pylori* stool antigen test (Meridian Bioscience Inc., Cincinnati, OH, USA). Although no study has validated this test in Thai children, most studies report its high sensitivity and specificity (>90%) (1).

The children enrolled in the study had a mean age of 7.9 years (range 0.5–16.6) and most were receiving both corticosteroids and chemotherapy (n = 36). Fourteen patients were being treated exclusively with corticosteroids, and 10 patients were receiving only chemotherapy. A total of 17.4% of the children <5 years of age had *H. pylori* infection, and the overall prevalence was 20%. Although we observed a relatively high prevalence of infection in patients with malignancy, particularly leukemia, the trend did not reach statistical significance (Table).

**Table:** *Helicobacter pylori* stool antigen test results in immunocompromised children and primary diagnosis*

Primary diagnosis	*H. pylori* stool antigen test	
	No. positive	No. negative
Malignancy		
Leukemia	8	21
Lymphoma	2	3
Neuroblastoma	0	7
Retinoblastoma	0	2
Nonmalignancy		
Nephrotic syndrome	1	8
SLE	0	6
Chronic renal failure	1	1

*SLE, systemic lupus erythematosus.

In contrast to previous studies that reported a low prevalence of infection with *H. pylori* in patients with AIDS (2) and leukemia (3), we demonstrated that its prevalence in immunocompromised Thai children (20%) was higher than that previously reported in a healthy Thai population (17.5%) (4) and in those with recurrent abdominal pain (11.3%) (5). The prevalence in children <5 years of age was high compared with that reported from Perez-Perez et al. (17.4% vs. 5%) (4). Although unintentional eradication of *H. pylori* after multiple courses of antimicrobial drugs in such patients could explain the low prevalence in some studies, commonly prescribed antimicrobial drugs without antisecretory agents may be unable to cure the infection.

The major limitations of this preliminary study were the use of different diagnostic methods in the various studies and the lack of healthy controls. Thus, a well-designed case-control study is needed. However, the prevalence of infection with *H. pylori* in the immunocompromised children was high, and these patients appear to be more susceptible to this infection in early life.
